# The importance of electrical parameters on transcutaneous tibial nerve stimulation for overactive bladder syndrome: a systematic review and meta-analysis

**DOI:** 10.1093/ageing/afaf203

**Published:** 2025-07-25

**Authors:** David Alejandro Vaca-Benavides, Wei Ju, Camila Gonzalez, Peter Aitken, Anil Kumar Appukuttan Nair Syamala Amma, Srinjoy Mitra, Susan D Shenkin

**Affiliations:** Advanced Care Research Centre, The University of Edinburgh Usher Institute of Population Health Sciences and Informatics, Edinburgh, Scotland, UK; Institute for Integrated Micro and Nano Systems, The University of Edinburgh School of Engineering, Edinburgh, Scotland, UK; Institute for Integrated Micro and Nano Systems, The University of Edinburgh School of Engineering, Edinburgh, Scotland, UK; Department of Urology, Western General Hospital, Edinburgh, Scotland, UK; Department of Medicine of the Elderly, Royal Infirmary of Edinburgh, Edinburgh, Scotland, UK; Advanced Care Research Centre, The University of Edinburgh Usher Institute of Population Health Sciences and Informatics, Edinburgh, Scotland, UK; Institute for Integrated Micro and Nano Systems, The University of Edinburgh School of Engineering, Edinburgh, Scotland, UK; Advanced Care Research Centre, The University of Edinburgh Usher Institute of Population Health Sciences and Informatics, Edinburgh, Scotland, UK; Institute for Integrated Micro and Nano Systems, The University of Edinburgh School of Engineering, Edinburgh, Scotland, UK; Advanced Care Research Centre, The University of Edinburgh Usher Institute of Population Health Sciences and Informatics, Edinburgh, Scotland, UK; Institute for Integrated Micro and Nano Systems, The University of Edinburgh School of Engineering, Edinburgh, Scotland, UK

**Keywords:** tibial nerve stimulation, electrical parameters, overactive bladder, systematic review, meta-analysis, older people

## Abstract

**Introduction:**

Overactive Bladder Syndrome (OAB) is common and distressing for older people. Transcutaneous Tibial Nerve Stimulation (TTNS) is a potential treatment when others have failed. Different electrical parameters—electrical frequency, intensity, pulse width—are used. We investigated their effect on urinary incontinence, urgency, frequency and nocturia.

**Methods:**

We searched MEDLINE, EMBASE and AMED to March 2025 for studies comparing TTNS to control/sham in adults with OAB in clinical/home settings. Two reviewers independently screened papers, extracted data and assessed risk of bias (using RoB2). We summarised studies narratively and used the GRADE framework. We meta-analysed RCTs (random-effects), grouping by electrical parameters. PROSPERO registration CRD42024490465.

**Results:**

We included 42 papers (13 RCTs), involving 2715 participants (972 in RCTs). Nearly half studies (47.6%) included people aged > = 60. TTNS protocols varied: electrical frequency 10/20 Hz; intensity set with sensory or motor threshold; pulse width mostly 200 microseconds. No serious adverse effects were reported, only mild pain/discomfort. TTNS at 10 Hz improved incontinence episodes (MD = –1.24, 95%CI –2.09 to −0.39, n = 255, N = 3). TTNS with motor threshold improved urgency (MD = –1.44, 95%CI –2.69 to −0.19, n = 193, N = 4) and nocturia (MD = –1.14, 95%CI –1.93 to −0.34, n = 153, N = 3). Risk of bias was low.

**Conclusions:**

TTNS is a safe option to treat OAB. Electrical parameters may impact effectiveness (in favour of 10 Hz, motor threshold). Certainty is reduced due to the small number of studies and participants. Future studies should include different electrical parameters to clarify their impact, particularly with older people and to allow standardisation of future treatment and effect on specific symptoms.

## Key Points

- Transcutaneous Tibial Nerve Stimulation (TTNS) is a promising treatment for Overactive Bladder Syndrome symptoms, potentially suitable for self-management at home.
- TTNS at 10 Hz may improve urinary incontinence episodes.
- Motor threshold stimulation intensity may reduce urgency and nocturia.

## Introduction

Overactive Bladder Syndrome (OAB) includes urinary urgency, frequency and nocturia and in some cases incontinence [1]. Detrusor overactivity manifests in a subset of patients [2], and, in many, the underlying cause is unclear. OAB predominantly affects older people leading to an increased risk of falls, low quality of life, loneliness and stigma [3, 4]. Over 40% of men and 30% of women aged 75 years and older have OAB [5]. OAB Management consists of progressive interventions involving lifestyle changes, medications and surgery. However, these are not always tolerated or effective [6, 7].

Neuromodulation is an alternative approach involving a controlled electrical stimulation—by an implantable or transcutaneous stimulator—to nerves linked to bladder control, such as sacral, pudendal and tibial nerves. Transcutaneous Tibial Nerve Stimulation (TTNS) accesses the posterior tibial nerve at the ankle using a pair of surface electrodes. Previous reviews found a reduction of urinary symptoms [8] and improved quality of life [9]. Additionally, symptom improvement from its implantable version [10, 11] and beta-3 adrenergics [12] are similar. However, these had multiple comparators and did not report the influence of electrical parameters. Evidence in older people living with frailty is more limited, with the ELECtric trial in frail older people in care homes showing no clinical or statistically significant effect on urinary incontinence, but resident-related factors might have impacted this finding [13].

TTNS requires minimal user training and is potentially suitable for home use. Trials of TTNS have similar treatment duration (~12 weeks) and stimulation time per session (~30 minutes). However, the stimuli signal electrical parameters are variable. Three aspects need to be considered: electrical frequency, the number of times the signal is generated per second (unit: cycles per second or Hertz [Hz]); intensity (unit: amperes) and pulse width, time in which the signal is active during each cycle (unit: seconds). These parameters are related to the energy delivered, which may influence OAB symptoms [14]. Recent studies showed their impact with Sacral Nerve Modulation (SNM) in humans [15–17], however, studies investigating this effect with Tibial Nerve Stimulation (TNS) are limited to animal models [18–20]. Previous systematic reviews and meta-analyses assessed the effectiveness of TTNS in treating OAB, but none has investigated the effect of electrical parameters. The aim of this systematic review and meta-analysis is to summarise the influence of electrical signal parameters of TTNS on urinary incontinence, urgency, frequency and nocturia in adults with OAB.

## Methods

The systematic review and meta-analysis was conducted according to the protocol registered on PROSPERO (CRD42024490465), and based on the Cochrane Handbook for Systematic Reviews of Interventions [21]. It is reported using the Preferred Reporting Items for Systematic reviews and Meta-Analyses (PRISMA) statement [22] (Appendix 1).

### Search strategy and selection criteria

Systematic searches were conducted with MEDLINE, EMBASE and AMED databases on January 2024 and updated on March 2025. The search strategy was devised in collaboration with a librarian and comprised a combination of Medical SubHeading and in-text search of terms related to TTNS and OAB (Appendix 2). Articles written in languages other than English were translated using online translation services.

The inclusion criteria were: (i) adults aged over 18 years with idiopathic or neurogenic OAB, (ii) TTNS used to treat OAB in clinical or home settings, (iii) placebo, sub-threshold or sham stimulation, (iv) primary outcomes included self-reported urinary symptoms. Secondary outcomes comprised OAB scores, quality of life and adverse event reports. All study types were eligible for inclusion, apart from systematic reviews or protocols: randomised controlled trials (RCTs), feasibility and pilot trials, prospective studies and qualitative studies. Studies were excluded if they were conducted in children, adolescents or animals and if they included combined interventions, or comparison groups other than placebo or sub-threshold stimulation.

Results were imported to Covidence [23], deduplicated automatically and checked manually by one reviewer (DAVB). Two reviewers screened independently titles and abstracts (DAVB, WJ, CG, PA, AK and SDS) and full-text (DAVB, WJ, CG, PA and AK) against the inclusion criteria. Discrepancies were resolved by consensus.

Data from RCTs were extracted by two reviewers (DAVB, WJ and CG) with good agreement. For other studies, one reviewer (DAVB) extracted information. Data included number of participants (male/female), age, study design, intervention duration, setting, electrical parameters of stimulation (frequency, pulse width, intensity), outcome measures at baseline and post-intervention and adverse events.

### Synthesis

Data from all studies was synthesised with a narrative approach. Meta-analysis was conducted with data from RCTs with RevMan 8.20 using the inverse-variance method and random-effects models. Studies with missing data were excluded. Outcomes were presented as mean differences, standard deviations and associated forest plots. Combined means and standard deviations were calculated for studies reporting several intervention arms to produce pair-wise comparisons. Heterogeneity was examined using the chi-squared test (*P* < .10 deemed statistically significant, instead of *P* < .05, due to the small number of included studies and sample sizes). I^2^ statistic >50% represents substantial heterogeneity.

Subgroup analysis was conducted to explore differences in treatment outcomes due to variations of electrical parameters in the stimulation signal and to account for possible causes of heterogeneity. Interventions with multiple arms were grouped accordingly. For such cases, the number of participants in the control group were divided evenly with the same mean and standard deviation to avoid double-counting of participants and reduce unit-of-analysis errors. Publication bias analysis with funnel plots was not possible due to the small number of RCTs included.

Assessment of methodological quality of RCTs was conducted by two reviewers independently (DAVB and WJ) using the Cochrane Risk-of-Bias 2 (RoB2) assessment tool [24], evaluating five components: randomisation process, deviations from intended interventions, missing outcome data, outcome measurement and selection of the reported results. Low quality (high RoB) studies were not excluded as the aim was to perform a comprehensive analysis of available literature. Discrepancies were resolved by consensus. The certainty of the evidence included in the meta-analysis was evaluated using the GRADE approach [25].

## Results

**Figure 1:**
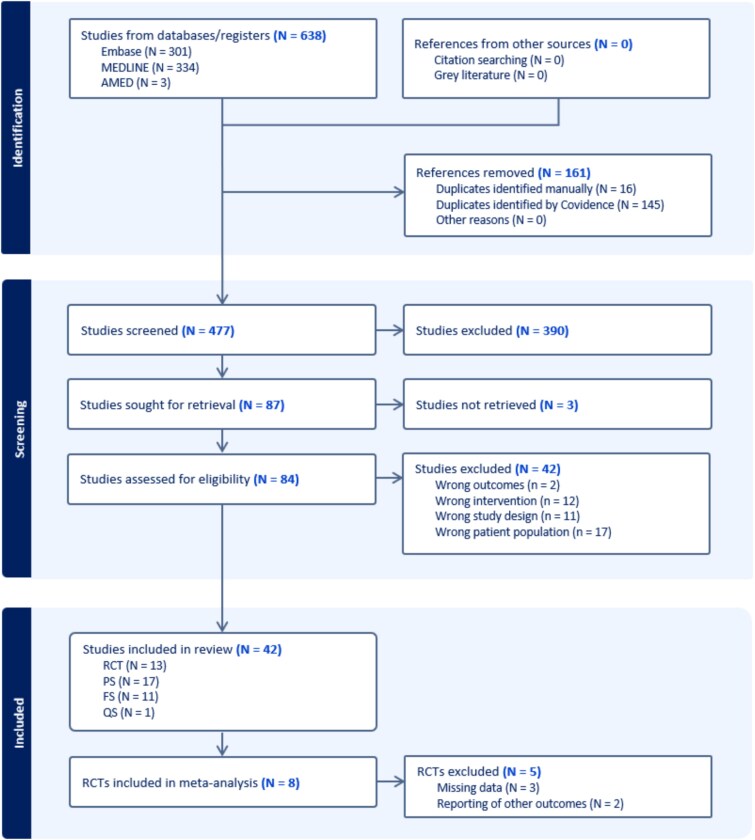
PRISMA Flowchart of study selection. (RCT: randomised controlled trials, PS: prospective studies, FS: feasibility studies, QS: qualitative studies).

The search strategy is summarised in Figure 1. Most studies were excluded because of combined interventions with TTNS. In total, 42 papers were included: 13 RCTs [26–38], 11 feasibility studies [39–49], 17 Prospective studies [50–66] and one qualitative study [67] (Table 1).

**Table 1:** Characteristics of included studies

Author year country	Age (SD) [Range]	Study design	Type of OAB	Participants (female/men)	Intervention/comparison	Setting	Duration (weeks)	Sessions per week	Session duration (minutes)	Stim Freq (Hz)	Pulse Width (μs)	Intensity	Outcomes
Bellette et al. 2009 Brazil [26]	47.73 (10.9)	RCT	Idiopathic	Int: 21 (21/0)	TTNS with Dualpex 961 device.	Hospital	4	2	30	NR	NR	NR	Three-day voiding diary OAB-q
				Cont: 16 (16/0)	Sham stimulation								
Cava and Orlin 2022 US [27]	64.4 (6.2)	RCT	Idiopathic	Int: 21 (18/3)	TTNS with ZIDA—a device for home use with textile electrodes.	Home	12	1	30	20	200	Motor threshold	Three-day voiding diary OAB-q SF
	72.7 (8.5)			Cont: 19 (14/5)	Sham stimulation								
Monteiro et al. 2014 Brazil [28]	65.1 (3.6)	RCT	Neurogenic	Int: 12 (0/12)	TTNS with Dualpex 961 device.	Hospital	6	2	30	10	200	Sensory threshold	Three-day voiding diary SISQ
	56.1 (10.9)			Cont: 12 (0/12)	Placebo								
Pierre et al. 2021 Brazil [29]	58 (9)	RCT	Idiopathic	Int1: 26 (26/0)	TTNS with Dualpex 961 device. Int1: once a week in one leg	Hospital	12	1	30	10	200	Sensory threshold	Three-day voiding diary OAB-V8
	62 (9)			Int2: 26 (26/0)	Int2: once a week in both legs	Hospital	12	1					
	62 (7)			Int3: 27 (27/0)	Int2: twice a week in one leg	Hospital	6	2					
	62 (10)			Int4: 28 (28/0)	Int3: twice a week in both legs	Hospital	6	2					
	60 (11)			Cont: 30 (30/0)	Placebo								
Teixeira Alve et al. 2020 Brazil [30]	67.52 (6.17)	RCT	Idiopathic	Int1: 33 (33/0)	TTNS at two different intensity levels. Int1: sensory threshold	Hospital		2	30	10	200	Sensory threshold	Three-day voiding diary ICIQ-OAB VAS (discomfort)
	69.57 (6.36)			Int2: 30 (30/0)	Int2: motor threshold	Hospital	4	2	30	10	200	Motor threshold	
	69.48 (7.83)			Cont: 25 (25/0)	No intervention								
Welk and McKibbon 2020 Canada [31]	64 [59–70]	RCT	Idiopathic	Int: 10 (10/0)	TTNS with EV-906 digital device.	First session in clinic with supervision, the rest at home	12	3	30	10	200	Motor threshold	PPBC 24-hour pad weights (g) Functional capacity (ml) PROM
	63 [58–67]			Cont: 10 (10/0)	Sham stimulation								
Araujo et al. 2021 Brazil [32]	64.2 (2.5)	RCT	Neurogenic	Int: 15 (15/0)	TTNS with custom device (developed by the Biomedical Engineering Service of the Hospital de Clínicas de Porto Alegre)	Home	12	5	20	10	200	Motor threshold	24-hour voiding diary OAB-V8 KHQ
	68.2 (2.3)			Cont: 15 (15/0)	Sham stimulation								
McClurg et al. 2022 UK [33]	68.7 (8.5)	RCT	Neurogenic	Int: 121 (47/74)	TTNS device and an instruction booklet were given to participants to take home.	Home	6	2	30	10	200	Motor threshold	ICIQ-SF IPSS Qualiveen questionnaire
	69.5 (8.7)			Cont: 121 (52/69)	Placebo								
Svihra et al. 2002 Slovakia [34]	54 [45–63]	RCT	Idiopathic	Int: 9 (9/0)	TTNS with myographic device Nicolet Viking II E. Patients were in supine position.	Hospital	5	1	30	1	100	Motor threshold	IPSS IQOL BUS
				Cont: 9 (9/0)	No intervention								
Goudelocke et al. 2024 USA [35]	62.7 (11.77)	RCT	Idiopathic	Int: 55	TTNS with Vivally System	Home	12	3	30	20	Variable	Set with EMG threshold	Three-day voiding diary OAB-q I-QOL OABSS PGII
				Cont: 52	Sham stimulation								
Zhang et al. 2025 China [36]	46.5 [21–72]	RCT	Idiopathic	Int: 54 (42/12)	TTNS with TNS-01 device	Home	4	3	30	NR	NR	Sensory threshold	Three-day voiding diary OABSS Maximum voided volume OAB-q IPSS
	49 [21–76]			Cont: 55 (43/12)	Sham stimulation								
Liao et al. 2024 China [37]	48.2 (15.6)	RCT	Idiopathic	Int: 43 (31/12)	TTNS with butterfly-shaped electrodes (General Stim)	Hospital	6	1	60	20	200	Motor threshold	Three-day voiding diary Maximum voided volume OABSS PPBC AUA-SI-QoL
	45.6 (14.1)			Cont: 42 (37/5)	Sham stimulation								
Galhardo et al. 2025 Brazil [38]	55.2 (14.7)	RCT	Idiopathic	Int1: 17 (17/0)	TTNS with Dualpex 961 device.	Hospital	6	2	30	10	200	Motor threshold	Three-day voiding diary ICIQ-OAB QUID IQOL PROMIS
	52.7 (12.3)			Int1: 18 (18/0)	Int2: bilateral TTNS								
Barnard et al. 2020 [39]	NR	FS	Idiopathic	40	TTNS with confirmation of nerve recruitment with EMG signals	NR	NR	NR	NR	NR	NR	NR	Evoked EMG
Bentellis et al. 2020 [40]	NR	FS	Neurogenic and idiopathic	134	TTNS with URO Stim 2 device. Participants were allocated in two groups. Int1: neurogenic OAB	Home	8	5	20	NR	NR	NR	Three-day voiding diary MHU Qualiveen questionnaire
					Int2: idiopathic OAB								
Li et al. 2022 China [41]	33.5 [29–59.75]	FS	Idiopathic	20 (12/8)	TTNS with General Stim device with butterfly-shape electrodes.	NR	4	5	60	20	200	Motor threshold	Three-day voiding diary OABSS AUA-SI-QOL PPBC
Li et al. 2023 China [42]	42.39 (19.72)	FS	Idiopathic	18 (18/0)	TTNS with General Stim device with butterfly-shape electrodes.	Hospital	4	5	60	20	200	NR	Three-day voiding diary OABSS AUA-SI-QOL PPBC
Maurelli et al. 2012 Italy [43]	49.6 (11.9)	FS	Idiopathic	16 (13/3)	TTNS with LogiSTIM device.	Home	12	Variable	30	20	200	NR	Three-day voiding diary IQOL
Ohannessian et al. 2013 France [44]	62 [56–71]	FS	Neurogenic	6 (6/0)	TTNS with URO Stim 2 device.	Home	6	5	20	10	0	Motor threshold	Three-day voiding diary PGII OAB-V8
Seth et al. 2018 UK [45]	46.4 [32–73]	FS	Neurogenic (50%), idiopathic (50%)	24 (18/6)	TTNS with GECKO-2 device. Participants were allocated in two groups. Int1: TTNS once daily	Home	12	5	30	1	70–560	Motor threshold	GRA ICIQ-OAB ICIQ-LUTSqol Three-day voiding diary
	46.9 [20–81]			24 (20/4)	Int2: TTNS once weekly								
Seth et al. 2015 UK [46]	NR	FS	Neurogenic (50%), idiopathic (50%)	20	TTNS with GECKO-2 device. Participants were allocated in two groups. Int1: TTNS once daily	NR	12	NR	30	NR	NR	NR	ICIQ-OAB ICIQ-LUTSqol Three-day voiding diary
				15	Int2: TTNS once weekly								
Slovak et al. 2015 UK [47]	59 (7.9)	FS	Idiopathic	19	TTNS with a conventional TENS machine. Participants were allocated in two groups. Int1: unilateral TTNS	Home	4	5	40	NR	NR	Sensory threshold	PGII Three-day voiding diary MCC
					Int2: bilateral TTNS								
Te Dorsthorst et al. 2021 Netherlands [48]	67 [36–86]	FS	Neurogenic (17%) and idiopathic (83%)	42 (34/8)	TTNS with Patients responders to PTNS.	Home	16 months [1–112]	Variable	NR	NR	NR	NR	Patient satisfaction and treatment score
Burton and Sokol 2024 USA [49]	70 [59–98]	FS	Idiopathic	18 (18/0)	TTNS with SoleStim device.	Home	8	3	30	20	NR	Motor threshold	Three-day voiding diary OAB-q
Alkis et al. 2021 Turkey [50]	51.04 (14.25)	PS	Idiopathic	30	TTNS with Neurotrac device and two different treatment periodicities. Int1: once a week	Hospital	12	1	30	10	200	0	OAB-V8 ICIQ-SF Urinary frequency Nocturia
	51.64 (13.52)			30	Int2: three times a week		12	3					
Ammi et al. 2014 France [51]	61.19 (15.73)	PS	Idiopathic	43 (36/7)	TTNS with URO Stim 2 device.	Home	4	5	20	10	no info	Motor threshold	USP MHU
de Seze et al. 2011 France [52]	48.3 (10.2)	PS	Neurogenic	70 (51/19)	TTNS with TENStem Eco device.	Home	12	5	20	10	200	Motor threshold	Three-day voiding diary MHU Qualiveen questionnaire VAS (Psychological and social burden)
Yildiz and Celtek 2023 Turkey [53]	62.05 (15.68)	PS	Idiopathic	21 (21/0)	TTNS with Enraf Nonius Myomed 932. Participants were allocated in two groups. Int1: TTNS with refractory group	Hospital	6	2	30	20	200	Motor threshold	Three-day voiding diary 24-h pad test OAB-V8
	59.14 (9.05)			21 (21/0)	Int2: TTNS with naïve group								
Moratalla Charcos et al. 2018 Spain [54]	66.6 (10.5)	PS	Idiopathic	45 (38/7)	TTNS with ELPHA II 3000 device. Participants were allocated in two groups. Int1: OAB with detrusor overactivity	Hospital	36	Variable	30		200	Motor threshold	Three-day voiding diary. Maximum voided volume OABq-SF
					Int2: OAB with no detrusor overactivity								
Charvolin et al. 2019 France [55]	65.59 (5.1)	PS	Neurogenic	17 (9/8)	TTNS with URO Stim 2 device.	Hospital	24	1–2	20	10	200	Sensory threshold	USP Qualiveen questionnaire
Blazejewski et al. 2010 [56]	NR	PS	Neurogenic	70 (51/19)	TTNS with measurement of tolerability parameters at day-30 and day-90	NR	NR	NR	NR	NR	NR	NR	Three-day voiding diary MHU Qualiveen questionnaire
Garate et al. 2019 Chile [57]	NR	PS	Idiopathic	126 (126/0)	TTNS with TENS 7000 device.	NR	6	2	30	20	200	NR	PGII OAB-q
Manriquez et al. 2022 Chile [58]	79 [68–90]	PS	Idiopathic	37 (37/0)	TTNS with short training beforehand.	Home	4	5	NR	NR	NR	NR	Three-day voiding diary OAB-Q
Booth et al. 2017 UK [59]	63.1 (15.2)	PS	Neurogenic (16%) and idiopathic	69 (60/9)	TTNS sessions at home, with the initial training at hospital, where a nurse gave training to participants of how to use the device.	Home	10	NR	NR	NR	NR	NR	IPSS ICIQ-SF
Hentzen et al. 2018 Germany [60]	74.1 (6.5)	PS	Neurogenic (53%) and idiopathic (47%)	264 (167/97)	TTNS with URO Stim 2 device in two centres Int1: TTNS at centre 1	Home	NR	5	20	15	150	NR	Global efficacy
					Int2: TTNS at centre 2			5	20	14	210	NR	
Kaga et al. 2023 Japan [61]	64.86 (17.98)	PS	Idiopathic	29 (9/20)	TTNS with SSP Alpha 1 electrodes for ‘accupunture-like’ stimulation.	NR	6	2	30	100	50	NR	OABSS ICIQ-SF Free uroflowmetry and frequency volume chart
Leroux et al. 2018 France [62]	58.4 (16.6)	PS	Neurogenic (94%) and idiopathic (6%)	103 (81/22)	TTNS with URO Stim 2 device.	Home	12	5	20	100	NR	Motor threshold	USP
Mass-Lindenbaum et al. 2019 Chile [63]	NR	PS	Idiopathic	120	TTNS at home or hospital.	Home and hospital	NR	NR	NR	NR	NR	NR	PGII
Peyronnet et al. 2020 France [64]	58.3	PS	Idiopathic	62	TTNS with URO Stim 2 device.	Home	12	5	20	NR	NR	NR	PGII Three-day voiding dairy USP
Cornu et al. 2023 France [65]	56.34 [43–68]	PS	Idiopathic	77 (64/13)	TTNS with TENSI+	Hospital	12	5	20	10	200	NR	Three-day voiding dairy USP OAB-q VAS (quality of life)
Hentzen et al. 2024 France [66]	47.1 (11.5)	PS	Neurogenic	82 (67/15)	TTNS with URO Stim 2 device.	Home	12	5	20	10	200	Sensory threshold	Three-day voiding dairy USP PGII Free uroflowmetry Cystometry
Daly et al. 2021 UK [67]	61 [38–78]	QS	Idiopathic	31 (31/0)	TTNS with Neurotrac device	Home	6	2	30	10	200	Motor threshold	Perceptions and expectations of TTNS management

AUA-SI-QOL: American Urological Association Symptom Index—Quality of Life; BUS: Behavioural Urge Score; EMG: Electromyography; FS: Feasibility Study; GRA: Global Rating of Improvement; HADS: Hospital Anxiety and Depression Scale; ICIQ-LUTSqol: International Consultation on Incontinence Questionnaire—Lower Urinary Tract Symptoms Quality of Life; ICIQ-OAB: International Consultation on Incontinence Questionnaire-Overactive Bladder; ICIQ-SF: International Consultation on Incontinence Questionnaire—Urinary Incontinence Short Form; IPSS: International Prostate Symptom Score; IQOL: Incontinence Quality of Life questionnaire; KHQ: King’s Health Questionnaire; MCC: Maximum Cystometric Capacity; MHU: Mesure du Handicap Urinaire; NR: Not Reported; OAB: Overactive Bladder; OABSS: Overactive Bladder Syndrome Score; OAB-q: Overactive Bladder Questionnaire; OAB-q SF: Overactive Bladder Questionnaire Short Form; OAB-V8: overactive bladder questionnaire-Validated 8; PGII: Perceived Global Impression of Improvement; PPBC: Patient Perception of Bladder Condition questionnaire; PROM: Patient Reported Outcome Measure; PS: Prospective Study; QS: Qualitative Study; RCT: Randomised Controlled Trial; SISQ: Subjective Improvement on Symptoms Questionnaire; USP: Urinary Symptom Profile; VAS: Visual Analogue Scale.

Most studies (N = 19) were conducted in Europe, 13 in the Americas, seven in Asia and three did not specify. No studies were found in Africa or Oceania. Papers were published between 2009 and 2025, with the majority (N = 35; 83.3%) in the past 10 years.

### Characteristics of studies

Baseline characteristics: The 42 included studies recruited 2715 people, mostly female (n = 1667), although in seven studies sex was not reported. The average age ranged from 33.5 to 79 years (not reported in six studies), with almost half of studies (N = 20; 47.6%) including participants aged > = 60 years. Studies were conducted in home settings (N = 22; 52.4%), in hospitals (N = 14; 33.3%) and five did not report. No study was performed in care homes.

Treatment dose: TTNS interventions were not standardised. Treatment duration ranged from four to 36 weeks (mean: 9.5, SD: 6.1), with 12 weeks being the most common (33.3%). The number of sessions varied from one to five per week (mean: 3.3, SD: 1.65), and session duration was 20 to 60 minutes, with 30 minutes for half of studies (50%). Treatment dose was partially or not reported by eight studies [39, 46, 48, 56, 58–60, 63].

Stimulation parameters: The electrical frequency ranged from 1 to 100 Hz, with fifteen studies using 10 Hz (35.7%) and 10 using 20 Hz (23.8%). Twenty studies (47.6%) reported a pulse width of 200 microseconds. Stimulation intensity was not reported numerically but as subjective sensory threshold from participants, or motor threshold (hallux flexion). Seven studies (16.7%) reported sensory threshold, eighteen studies (42.3%) motor threshold. Seventeen did not report intensity.

Outcomes: Symptom improvement was mostly measured with 3-day voiding diary. Additionally, validated tools to assess OAB and Lower Urinary Tract Symptoms (LUTS) comprised the OAB-q questionnaire [26, 35, 36, 49, 57, 58], OAB-q Short Form (SF) [27, 54], OAB-V8 questionnaire [29, 32, 44, 50, 53], International Consultation on Incontinence Questionnaire OAB Module (ICIQ-OAB) [30, 38, 45, 46] and Urinary Incontinence Short Form (ICIQ-UI-SF) [33, 50, 59, 61], Measurement of Urinary Handicap (MHU) score [40, 51, 52, 56], OAB Symptom Score (OABSS) [35–37, 41, 42, 61], Urinary Symptoms Profile (USP) questionnaire [51, 55, 62, 64–66], International Prostate Symptom Score (IPSS) [33, 34, 36, 59]. Quality of life was measured with the Qualiveen questionnaire [33, 40, 52, 55, 56], Incontinence Quality of Life (I-QoL) questionnaire [34, 38, 43], the American Urological Association Symptom Index Quality of Life Score (AUA-SI-QoL) [37, 41, 42], ICIQ-LUTS related Quality of Life score [45, 46] and King’s Health Questionnaire [32]. Other assessment tools used were the Patient Global Impression of Improvement (PGII) score [35, 44, 47, 57, 63, 64, 66] and Patient Perception of Bladder Condition (PPBC) questionnaire [31, 37, 41, 42].

### Narrative synthesis of results

All studies reported improvement in urinary symptoms in participants who received TTNS, whether as a reduction of 3-day voiding diaries measures (urinary incontinence, urgency, frequency and nocturia), or improved quality of life and OAB symptom scores. Results were presented as positive response rates, ranging from 15% to over 90% of participants. Some reported results of confidence intervals and P-tests. Symptoms improved in both neurogenic and idiopathic OAB groups, as well as OAB refractory to drug treatments [41, 42, 50–52, 57, 62, 64] or treatment-naïve [44, 53]. Patient perception was investigated by a few studies [27, 44, 48, 53–55], reporting good satisfaction levels. Treatment adherence was investigated by two studies, reporting a mean compliance of 8.3 [62] and 16 months [48], with the majority of drop-outs due to lack of sufficient symptom relief. Only one study [67] explored qualitatively the experiences of TTNS, presenting four themes related to consistency in managing bladder and perceptions of symptom improvement. There were no reports of serious adverse effects, with five studies [27, 30, 54, 55, 61] reporting mild side effects related to discomfort in the stimulation area.

### Characteristics of RCTs

Baseline characteristics: Thirteen RCTs recruited 972 participants (66.8% women), with 566 people allocated in the TTNS group and 406 in control groups (sham n = 209 [26, 27, 31, 32, 35–38], placebo n = 163 [28, 29, 33], no intervention n = 34 [30, 34]). Ten RCTs recruited participants with idiopathic OAB and three with neurogenic OAB (secondary to ischemic stroke [28] and Parkinson’s Disease [32, 33]). All RCTs compared one TTNS intervention arm against control groups, except for three RCTs. Pierre et al. [29] and Galhardo et al. [38] explored the effect of unilateral and bilateral TTNS. The two-arm intervention by Teixeira et al. [30] reported TTNS with intensity at sensory and motor threshold.

Treatment dose: Treatment duration was 4 to 12 weeks, one to five sessions per week, mostly for 30 minutes (N = 11).

Stimulation parameters: The electrical frequency used by seven RCTs was 10 Hz, and three reported 20 Hz. Seven studies set intensity as motor threshold, sensory threshold by three [28, 29], and one used both in different intervention arms [30]. Two RCTs did not report any stimulation parameter [26, 36]. The pulse width applied by eight RCTs was 200 microseconds, one used 100 microseconds [34] and one was variable [35].

Outcomes: All studies measured OAB or quality of life scores. Eleven RCTs collected data with 3-day voiding diaries [26–32, 35–38].

### Methodological quality of RCTs and certainty of evidence

**Figure 2:**
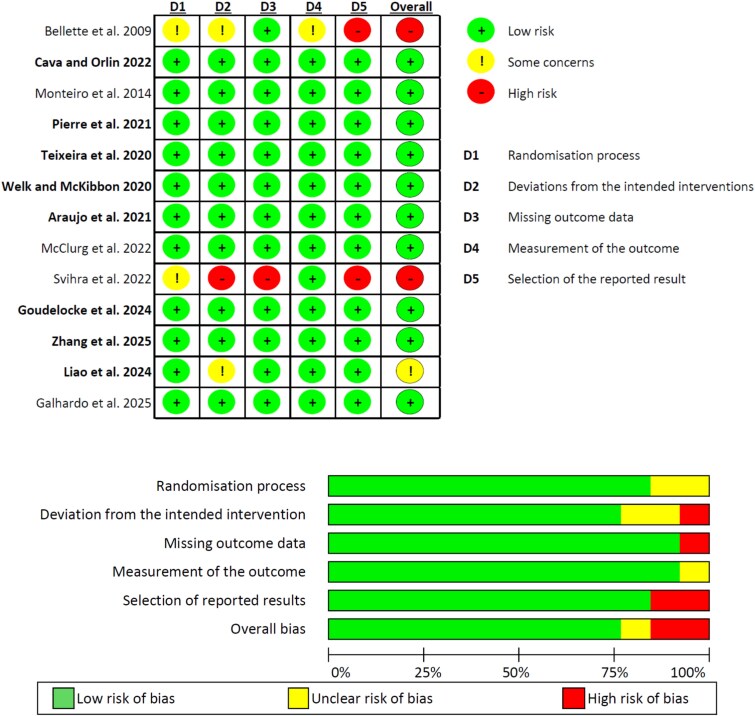
Risk-of-bias (RoB2) assessment. Studies included in the meta-analysis are indicated in bold.

The summary of the quality assessment for RCTs is presented in Figure 2, and full assessment in supplementary material (Appendices 3 and 4). Ten RCTs were evaluated with low risk-of-bias in all domains, one with unclear risk [37], and two [26, 34] with high risk. Common sources of bias were the randomisation process, deviation from the intended intervention and the selection of reported results.

Certainty of evidence ranged from low to high (Appendices 4 and 5). A minimal overlapping of CI between studies within subgroups was deemed as a serious case of inconsistency, and no overlapping as very serious inconsistency.

### Meta-analysis

Eight RCTs were included in the meta-analysis. Five were excluded: three due to missing data [26, 28, 38], and two because reported different outcome measures [33, 34]. The outcomes included in the meta-analysis were: average 24-hour episodes of urinary incontinence, urgency, frequency and nocturia. The studies were subgrouped by electrical frequency and stimulation intensity. A pulse width subgroup analysis was not possible as studies used 200 microseconds. All studies were assessed as low risk-of-bias except one [37], with unclear risk-of-bias in one category.

### Subgroup analysis of electrical frequency

The forest plots in Figure 3 show the subgroups of electrical frequency for urinary incontinence (3A), urgency (3B), urinary frequency (3C) and nocturia (3D). Results suggest a reduction in urinary incontinence episodes in favour of the TTNS group versus control (MD: –0.88, 95%CI: −1.58, −0.18, I^2^ = 77%). Results in the 10 Hz group show an improvement in this outcome (MD: –1.24, 95%CI: −2.09, −0.39, I^2^ = 70%), whereas 20 Hz has little or no effect (MD: –0.30, 95%CI: −0.63, 0.03, I^2^ = 0%). In both cases CIs overlapping suggests high-certainty evidence. The P-test for subgroups confirms the differences between them (*P* = .04).

**Figure 3:**
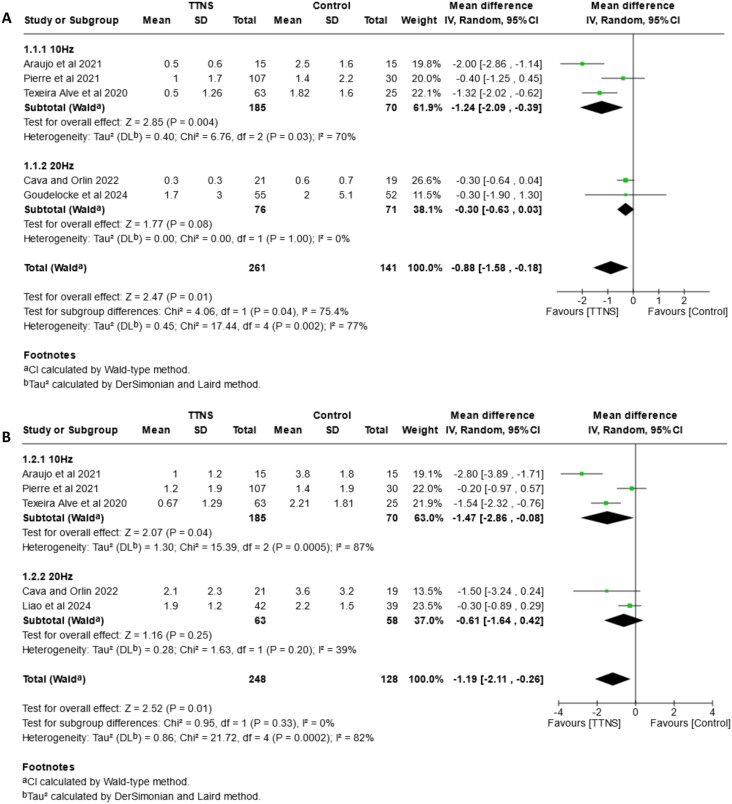
Forest plots—comparison of TTNS versus control, subgrouped by **electrical frequency (10 Hz and 20 Hz).** (A) effects on urinary incontinence, (B) effects on urgency, (C) effects on frequency (D) effects on nocturia.

**Figure 3:**
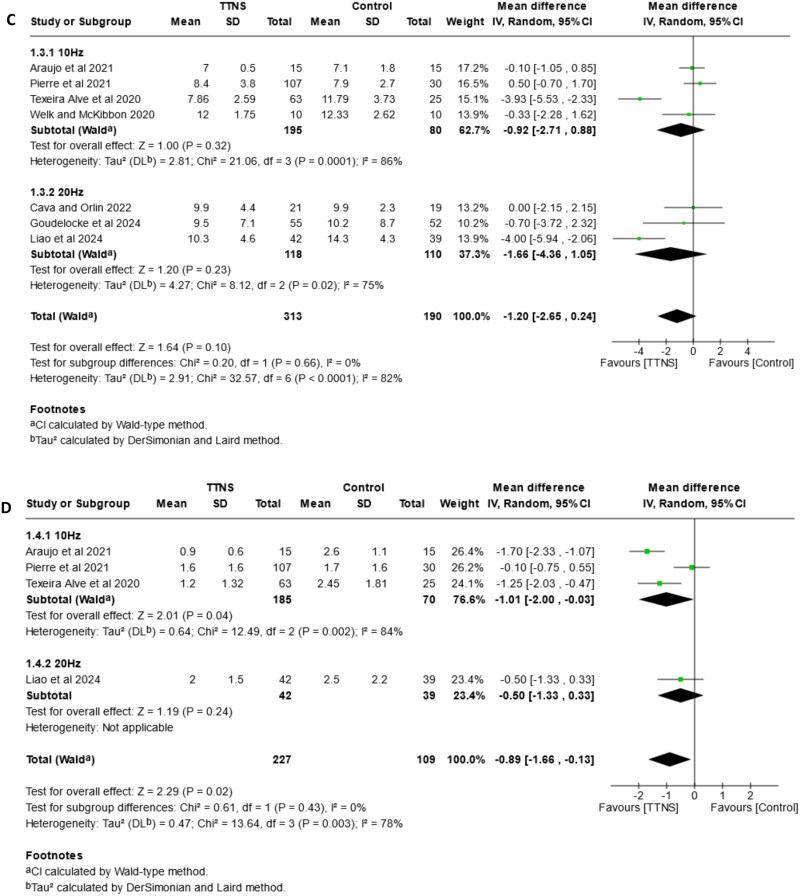
Continued.

Regarding urgency, there is a greater total effect towards the TTNS group (MD: –1.19, 95%CI: −2.11, −0.26, I^2^ = 82%), with a marginal reduction in urgency episodes in favour of 10 Hz (MD: –1.47, 95%CI: −2.86, −0.08, I^2^ = 87%). Results in the 20 Hz group do not indicate a reduction of urgency (MD: –0.61, 95%CI: −1.64, 0.42, I^2^ = 39%). Subgroup P-test shows no differences (*P* = .33).

For urinary frequency, there is no suggestion of overall improvement in favour of any group (MD: –1.20, 95%CI: −2.65, 0.24, I^2^: 82%). Subgroup analysis shows no differences (*P* = .66) between the 10 Hz (MD: –0.92, 95%CI: −2.71, 0.9, I^2^ = 86%) and 20 Hz groups (MD: –1.66, 95%CI: −4.36, 1.05, I^2^ = 75%), probably with little or no effect.

For nocturia results suggest improvement towards TTNS (MD: –0.89, 95%CI: −1.66, −0.13, I^2^ = 78%), with probably a slight improvement in favour of 10 Hz (MD: –1.01, 95%CI: −2.00, −0.03, I^2^ = 84%), and 20 Hz may have little or no improvement (MD: –0.50, 95%CI: −1.33, 0.33). The P-test shows no differences between groups (*P* = .43).

### Subgroup analysis of stimulation intensity

The forest plots in Figure 4 show the subgroup analysis of stimulation intensity for urinary incontinence (4A), urgency (4B), urinary frequency (4C) and nocturia (4D). One study [30] included two arms, with sensory and motor threshold respectively. Data was allocated in the correspondent group to enable meta-analysis.

**Figure 4:**
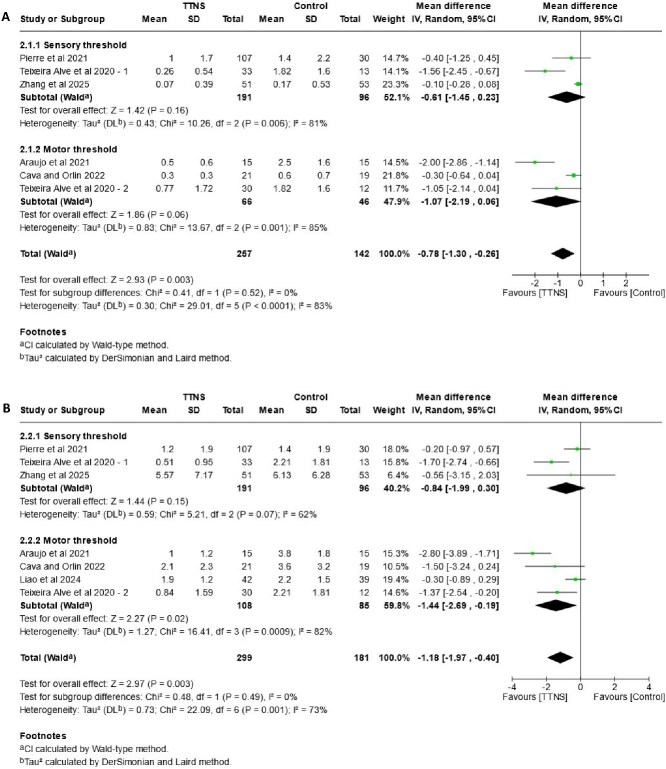
Forest plots—comparison of TTNS versus control, subgrouped by **stimulation intensity (sensory and motor threshold)**. (A) effects on urinary incontinence, (B) effects on urgency, (C) effects on frequency (D) effects on nocturia.

**Figure 4:**
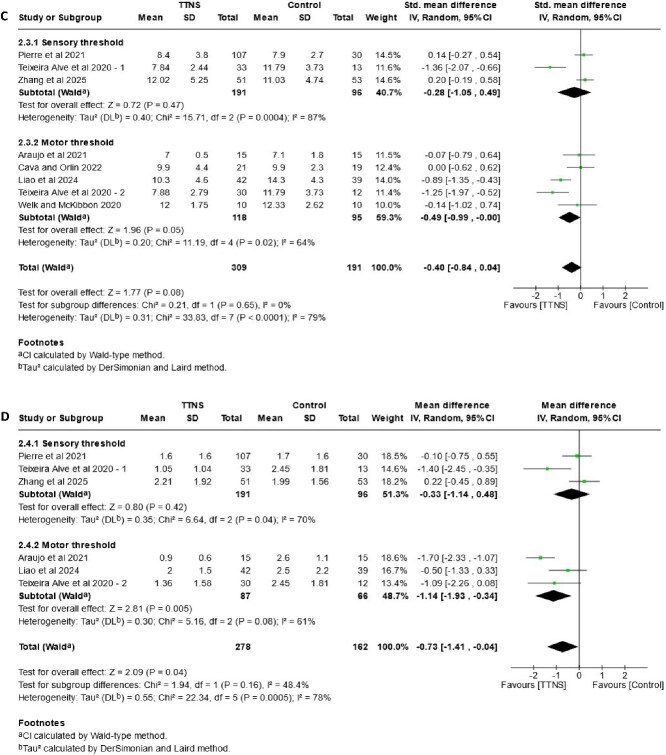
Continued.

An effect in favour of TTNS group is seen for urinary incontinence events (MD: –0.78, 95%CI: −1.30, −0.26, I^2^ = 83%), however, results from individual groups differ. The sensory threshold group (MD: –0.61, 95%CI: −1.45, 0.23, I^2^ = 81%) shows no difference against control, with moderate-certainty evidence. Results in the motor threshold group (MD: –1.07, 95%CI: −2.19, 0.06, I^2^ = 85%) indicate TTNS may have little or no effect. Subgroup test shows no differences between groups (*P* = .52).

The overall analysis of urinary urgency shows an effect in favour of the TTNS group (MD: –1.18, 95%CI: −1.97, −0.40, I^2^ = 73%). Results for motor threshold indicate a possible slight reduction of episodes (MD: –1.44, 95% CI: −2.69, −0.19, I^2^ = 82%), supported by moderate-certainty of evidence. On the other hand, results for the sensory threshold group (MD: –0.84, 95%CI: −1.99, 0.30, I^2^ = 62%), with high-certainty of evidence and a reduction of heterogeneity, show that there is little or no effect. Despite of this, the P-value for subgroups shows no significant differences (*P* = .49).

The analysis on urinary frequency shows no overall effect in favour of any group (MD: –0.40, 95%CI: −0.84, 0.04, I^2^ = 79%). Results for sensory threshold (MD: –0.28, 95%CI: −1.05, 0.49, I^2^: 87%) probably indicate no effect, while for motor threshold (MD: –0.49, 95%CI: −0.99, 0.00, I^2^ = 64%) there is little or no effect, with high certainty of evidence for this group. P-test of subgroup reveals no differences (*P* = .65).

For nocturia there is an overall effect in favour of TTNS (MD: –0.73, 95%CI: −1.41, −0.04, I^2^ = 78%), with improvement in the motor threshold group (MD: –1.14, 95%CI: −1.93, −0.34, I^2^ = 61%). In contrast, results for the sensory threshold group show little or no effect (MD: –0.33, 95%CI: −1.14, 0.48, I^2^ = 70%). In both cases, certainty of evidence is high. However, the P-value for subgroups shows no significant differences (*P* = .16).

## Discussion

In 35 studies (10 RCTs) OAB (idiopathic and neurogenic) symptoms improved in participants that underwent a TTNS programme. Participants were mostly older (> = 60 years) adults, both those who had symptoms refractory to anticholinergics and treatment-naïve. Electrical parameters were reported by most studies. Interventions were not standardised, with differences in electrical parameters and dose. Studies agreed in using a low-frequency (10/20 Hz) pulse signal. Stimulation intensity was not reported as electrical current, despite the capability of most stimulators, and was determined as a subjective sensory feeling, or tangible motor response. Pulse width varied slightly between studies (100–200 microseconds).

Our results indicate an overall improvement of urinary incontinence, urgency and nocturia episodes in favour of TTNS, but little or no improvement in urinary frequency. The meta-analysis included studies with TTNS as the only intervention, avoiding confounders from other treatments. All but one RCTs had low risk of bias. In the electrical frequency subgroup analysis, high-certainty of evidence and statistical significance of results indicate that urinary incontinence improves with TTNS using 10 Hz. In the case of urgency, urinary frequency and nocturia, evidence show moderate certainty regarding whether or not there is an effect with 10 Hz. Additionally, results indicate no improvement of incontinence and urgency with TTNS using 20 Hz, with high-certainty of evidence. For frequency and nocturia limited certainty of evidence precludes any confirmation of no effect. A reduction of heterogeneity in the 20 Hz group and incontinence group suggests a possible influence of electrical frequency.

Regarding stimulation intensity, high-certainty data indicates improvement of nocturia, but no effect in frequency for the TTNS group with motor threshold, and no improvement for nocturia in the sensory threshold group. Additionally, moderate-certainty evidence shows a possible improvement of urgency for TTNS with motor threshold. On the other hand, moderate certainty evidence indicates probably no effect for urinary incontinence in the motor and sensory threshold groups, and for urgency and frequency in the sensory threshold group. A reduction of statistical heterogeneity in the motor threshold group for nocturia, suggests that a higher intensity might influence this.

From an engineering perspective, the energy transported by the stimulation signal is proportional to the pulse width, frequency and intensity squared. Neuromodulation devices allow setting up electrical frequency, intensity and pulse width. However, there is no agreement across treatment programmes, potentially causing differences in symptom improvement. Initial studies involving neuromodulation of other nerves found promising results with maximum stimulation intensity [68, 69]. Studies targeting the pudendal and sacral nerves found that the degree of bladder inhibition in cats is strongly related to intensity [70], and in humans, the improvement in voiding diary data with SNM is related to a higher amplitudes [15, 71]. There are no studies of TNS in humans exploring these effects, however, trials with animal models show that increasing intensity is associated with recruitment of more bladder-inhibitory nerves [19]. Recent SNM techniques explore the effects of different frequencies to improve urinary symptoms [17], the impact of varying electrical frequency [72] and burst patterns [16], indicating that electrical frequency may mediate symptoms. Studies of TNS in animal models with low and high frequencies confirm that may influence the effects [18, 20]. Regarding pulse width, studies of SNM in humans indicate little or no effect on symptoms [15, 73, 74], but do preserve battery-life of implanted devices. There are no studies of TNS exploring the effect of pulse width. These findings show the need for further interdisciplinary research in this area.

This systematic review included broad search terms, dual data extraction, robust risk of bias assessment and use of the GRADE framework for data summary but results should be interpreted with caution. The number of studies, from those eligible for inclusion, which were RCTs, and of those, RCTs that could be included in the meta-analysis were small, and few included people in very old age, or living with significant frailty. Many of the trials did not report an age or OAB severity comparison between groups. Symptom reduction was described mainly with 3-day voiding diaries, OAB scores and quality of life assessments. However, data is mostly reported as percentages of positive responders, with a limited number of studies presenting confidence intervals, interquartile ranges or statistical significance tests. Subgroup analysis of OAB type was not possible as data was not stratified. The certainty of evidence was not classified as high in all cases, and there was high heterogeneity probably due to differences in protocols (duration, electrical parameters and settings), symptom severity and the combination of data from studies with multiple arms to enable the meta-analysis.

Another limitation is the lack of discussion of how TTNS may interact with comorbidities associated with OAB in older adults and other related problems such as urinary tract infections (UTI), risk of falls and fractures [75], cognitive decline [76] and frailty [77, 78]. Outcome measures are mainly focused on continence parameters. Although some also included health-related quality of life scores, a few assessed the psychosocial impact of OAB in patients, and even less frequently its burden on carers [79]. Moreover, studies excluded participants with UTI and cognitive decline, the latter potentially leading to an underrepresentation of the oldest-old adults in clinical trials. Settings were limited to home and hospitals, with no studies in care homes, where OAB is prevalent. The landmark ELECtric TTNS trial in care homes [13] was excluded from our review because they included people diagnosed with urinary incontinence and not OAB. Finally, although no serious adverse events were reported, studies did not discuss devices’ features, such as wearability and ease of use, and their impact on treatment uptake.

## Conclusion

TTNS is an attractive treatment for OAB symptoms, which is safe, and potentially suitable for self-management at home. Studies to date including older adults show some improvement in symptoms, and that electrical parameters may influence the outcomes. However, more studies are needed, including older people with a range of co-morbidities, and clearly reporting how electrical parameters influence clinically relevant outcomes.

## Supplementary Material

Amended_Supplementary_material_afaf203

Supplementary_material_afaf203_File002

Supplementary_material_afaf203_File003

Supplementary_material_afaf203_File004

Supplementary_material_afaf203_File005

Supplementary_material_afaf203_File006
