# Supplementary material for: The importance of electrical parameters on transcutaneous tibial nerve stimulation for overactive bladder syndrome: a systematic review and meta-analysis
Source: Age Ageing. 2025 Jul 25;54(7):afaf203. doi: 10.1093/ageing/afaf203 (PMC12291541; doi:10.1093/ageing/afaf203)
Supplement: Amended_Supplementary_material_afaf203 [file amended_supplementary_material_afaf203.zip › Supplementary_material_afaf203_File007.pdf]

Summary of findings:

Appendix 6: Certainty of Evidence - Stimulation Intensity Subgroup Meta-Analysis Title: The importance of electrical parameters on transcutaneous tibial nerve stimulation for overactive bladder syndrome: a systematic review and meta-analysis

Patient or population: Adults with Overactive Bladder Syndrome (OAB)
Setting: Home/Hospital
Intervention: Transcutaneous Tibial Nerve Stimulation (TTNS)
Comparison: Control

Outcomes	Anticipated absolute effects* (95% CI)		Relative effect (95% CI)	№ of participants (studies)	Certainty of the evidence (GRADE)	Comments
	**Risk with Control**	**Risk with Transcutaneous Tibial Nerve Stimulation (TTNS)**				
Urinary Incontinence (24h) - Sensory threshold	The mean urinary Incontinence (24h) - Sensory threshold was **0.96** episodes[a]	MD **0.62 episodes lower** (1.46 lower to 0.22 higher)	-	287 (3 RCTs)	⨁⨁⨁◯ Moderate[b]	
Urinary Incontinence (24h) - Motor threshold	The mean urinary Incontinence (24h) - Motor threshold was **1.47** episodes[a]	MD **1.07 episodes lower** (2.19 lower to 0.06 higher)	-	112 (3 RCTs)	⨁⨁⨁◯ Moderate[b]	
Urgency (24h) - Sensory threshold	The mean urgency (24h) - Sensory threshold was **2.48** episodes[a]	MD **0.85 episodes lower** (1.98 lower to 0.27 higher)	-	287 (3 RCTs)	⨁⨁⨁⨁ High	
Urgency (24h) - Motor threshold	The mean urgency (24h) - Motor threshold was **2.85** episodes[a]	MD **1.44 episodes lower** (2.69 lower to 0.19 lower)	-	193 (4 RCTs)	⨁⨁⨁◯ Moderate[b]	
Urinary Frequency (24h) - Sensory threshold	The mean urinary Frequency (24h) - Sensory threshold was **10.13** episodes[a]	MD **0.28 episodes lower** (1.05 lower to 0.49 higher)	-	287 (3 RCTs)	⨁⨁⨁◯ Moderate[b]	
Urinary Frequency (24h) - Motor threshold	The mean urinary Frequency (24h) - Motor threshold was **11.16** episodes[a]	MD **0.49 episodes lower** (0.99 lower to 0)	-	213 (5 RCTs)	⨁⨁⨁⨁ High	
Nocturia (24h) - Sensory threshold	The mean nocturia (24h) - Sensory threshold was **2.02** episodes[a]	MD **0.34 episodes lower** (1.15 lower to 0.47 higher)	-	287 (3 RCTs)	⨁⨁⨁⨁ High	
Nocturia (24h) - Motor threshold	The mean nocturia (24h) - Motor threshold was **2.52** episodes[a]	MD **1.14 episodes lower** (1.93 lower to 0.34 lower)	-	153 (3 RCTs)	⨁⨁⨁⨁ High	

***The risk in the intervention group** (and its 95% confidence interval) is based on the assumed risk in the comparison group and the **relative effect** of the intervention (and its 95% CI).

CI: confidence interval; **MD:** mean difference

GRADE Working Group grades of evidence
High certainty: we are very confident that the true effect lies close to that of the estimate of the effect.
Moderate certainty: we are moderately confident in the effect estimate: the true effect is likely to be close to the estimate of the effect, but there is a possibility that it is substantially different.
Low certainty: our confidence in the effect estimate is limited: the true effect may be substantially different from the estimate of the effect.
Very low certainty: we have very little confidence in the effect estimate: the true effect is likely to be substantially different from the estimate of effect.

Explanations

[a]Control group results measured at end-of-treatment and effect sizes were used to calculate mean scores.
[b]There is a partial overlap of confidence intervals, affecting the consistency (serious inconsistency).
