# Supplementary material for: The importance of electrical parameters on transcutaneous tibial nerve stimulation for overactive bladder syndrome: a systematic review and meta-analysis
Source: Age Ageing. 2025 Jul 25;54(7):afaf203. doi: 10.1093/ageing/afaf203 (PMC12291541; doi:10.1093/ageing/afaf203)
Supplement: Supplementary_material_afaf203_File003 [file supplementary_material_afaf203_file003.pdf]

Appendix 2: Search Strategy

Title: The importance of Electrical Parameters on Transcutaneous Tibial Nerve Stimulation (TTNS) for Overactive Bladder Syndrome: A Systematic Review and Meta-Analysis

Databases: MEDLINE, EMBASE, AMED

1.	(Urinary Incontinence/ or Nocturia/ or Urinary Bladder, Neurogenic/ or Urinary Bladder, Overactive/) and (Transcutaneous Electric Nerve Stimulation/ or Electric Stimulation Therapy/) and (tibial nerve/ or "TTNS".mp. or "TPTNS".mp.) [mp=ab, hw, ti, tn, ot, dm, mf, dv, kf, fx, dq, bt, nm, ox, px, rx, ui, sy, ux, mx]
2.	(("urinary incont*" or "urinary blad*" or "overactive blad*") and ("transcutaneous elec*" or "transcutaneous tibi*" or "transcutaneous ner*" or "TTNS" or "TPTNS") and ("tibial nerv*" or "posterior tib*")).mp. [mp=ab, hw, ti, tn, ot, dm, mf, dv, kf, fx, dq, bt, nm, ox, px, rx, ui, sy, ux, mx]
3.	1 or 2
