# Supplementary material for: The importance of electrical parameters on transcutaneous tibial nerve stimulation for overactive bladder syndrome: a systematic review and meta-analysis
Source: Age Ageing. 2025 Jul 25;54(7):afaf203. doi: 10.1093/ageing/afaf203 (PMC12291541; doi:10.1093/ageing/afaf203)
Supplement: Supplementary_material_afaf203_File006 [file supplementary_material_afaf203_file006.pdf]

Summary of findings:

Appendix 5: Certainty of Evidence - Electrical Frequency (Hz) Subgroup Meta-Analysis Title: The importance of Electrical Parameters on Transcutaneous Tibial Nerve Stimulation (TTNS) for Overactive Bladder Syndrome: A Systematic Review and Meta-Analysis

Patient or population: Adults with Overactive Bladder Syndrome (OAB)
Setting: Home/Hospital
Intervention: Transcutaneous Tibial Nerve Stimulation (TTNS)
Comparison: Control

Outcomes	**Anticipated absolute effects*** (95% CI)		Relative effect (95% CI)	№ of participants (studies)	Certainty of the evidence (GRADE)	Comments
	**Risk with Control**	**Risk with Transcutaneous Tibial Nerve Stimulation (TTNS)**				
Urinary Incontinence (24h) - 10Hz	The mean urinary Incontinence (24h) - 10Hz was **1.9** episodes[a]	MD **1.25 episodes lower** (2.09 lower to 0.41 lower)	-	255 (3 RCTs)	⨁⨁⨁⨁ High	
Urinary Incontinence (24h) - 20Hz	The mean urinary Incontinence (24h) - 20Hz was **1.02** episodes[a]	MD **0.3 episodes lower** (0.63 lower to 0.03 higher)	-	147 (2 RCTs)	⨁⨁⨁⨁ High	
Urgency (24h) - 10Hz	The mean urgency (24h) - 10Hz was **2.41** episodes[a]	MD **1.48 episodes lower** (2.85 lower to 0.1 lower)	-	255 (3 RCTs)	⨁⨁⨁◯ Moderate[b]	
Urgency (24h) - 20Hz	The mean urgency (24h) - 20Hz was **2.71** episodes[a]	MD **0.61 episodes lower** (1.64 lower to 0.42 higher)	-	121 (2 RCTs)	⨁⨁⨁⨁ High	
Urinary Frequency (24h) - 10Hz	The mean urinary Frequency (24h) - 10Hz was **9.55** episodes[a]	MD **0.91 episodes lower** (2.71 lower to 0.9 higher)	-	275 (4 RCTs)	⨁⨁⨁◯ Moderate[b]	
Urinary Frequency (24h) - 20Hz	The mean urinary Frequency (24h) - 20Hz was **11.62** episodes[a]	MD **1.66 episodes lower** (4.36 lower to 1.05 higher)	-	228 (3 RCTs)	⨁⨁⨁◯ Moderate[b]	
Nocturia (24h) - 10Hz	The mean nocturia (24h) - 10Hz was **2.25** episodes[a]	MD **1.02 episodes lower** (1.99 lower to 0.05 lower)	-	255 (3 RCTs)	⨁⨁⨁◯ Moderate[b]	
Nocturia (24h) - 20Hz	The mean nocturia (24h) - 20Hz was **2.5** episodes[a]	MD **0.5 episodes lower** (1.33 lower to 0.33 higher)	-	81 (1 RCT)	⨁⨁◯◯ Low[c]	

***The risk in the intervention group** (and its 95% confidence interval) is based on the assumed risk in the comparison group and the **relative effect** of the intervention (and its 95% CI).

CI: confidence interval; **MD:** mean difference

GRADE Working Group grades of evidence
High certainty: we are very confident that the true effect lies close to that of the estimate of the effect.
Moderate certainty: we are moderately confident in the effect estimate: the true effect is likely to be close to the estimate of the effect, but there is a possibility that it is substantially different.
Low certainty: our confidence in the effect estimate is limited: the true effect may be substantially different from the estimate of the effect.
Very low certainty: we have very little confidence in the effect estimate: the true effect is likely to be substantially different from the estimate of effect.

Explanations

[a] Control group results measured at end-of-treatment and effect sizes were used to calculate mean scores.
[b] There is a partial overlap of confidence intervals, affecting the consistency (serious inconsistency).
[c] There is only one study for one of the subgroups, affecting the precision (very serious imprecision)
